# Divergent EGFR/MAPK-Mediated Immune Responses to Clinical *Candida* Pathogens in Vulvovaginal Candidiasis

**DOI:** 10.3389/fimmu.2022.894069

**Published:** 2022-05-26

**Authors:** Jingyun Zhang, Jingwen Peng, Dongmei Li, Huan Mei, Yu Yu, Xiaofang Li, Xiaodong She, Weida Liu

**Affiliations:** ^1^ Department of Medical Mycology, Institute of Dermatology, Chinese Academy of Medical Sciences (CAMS) & Peking Union Medical College (PUMC), Nanjing, China; ^2^ Jiangsu Key Laboratory of Molecular Biology for Skin Diseases and STIs, Nanjing, China; ^3^ Department of Microbiology & Immunology, Georgetown University Medical Center, Washington, DC, United States; ^4^ Center for Global Health, School of Public Health, Nanjing Medical University, Nanjing, China

**Keywords:** *Candida albicans*, non-albicans *Candida* species, vaginal epithelial cells, EGFR, MAPK, vulvovaginal candidiasis (VVC)

## Abstract

Vulvovaginal candidiasis (VVC) is characterized by symptomatic inflammatory responses in the vagina caused by *Candida albicans* and non-albicans *Candida* (NAC) species. The epidermal growth factor receptor (EGFR) -mitogen-activated protein kinase (MAPK) signaling pathway has been linked to immune responses of oral mucosa after *C. albicans* exposure, but whether this pathway plays a similar response in vaginal epithelial cells is not known. Here, we observed that phosphorylation of EGFR and p38 was continuously activated in vaginal epithelial cells by *C. albicans* strain SC5314. This differs markedly from oral epithelial cells, which respond in a biphasic manner in order to properly discriminate the morphology of *C. albicans*. When compared with SC5314, a highly azole-resistant *C. albicans* isolate 1052 can induce a stronger phosphorylated signal of EGFR and p38, while clinically-isolated NAC strains including *C. tropicalis*, *C. glabrata*, *C. parapsilosis* and *C. auris* trigger higher levels of phosphorylated ERK1/2 and c-Fos than *C. albicans*. Inhibition of EGFR significantly reduces inflammatory response and epithelial damage induced by *C. albicans* both *in vitro* and *in vivo*, while inhibition of p38 leads to significant repair of epithelial damage triggered by both *C. albicans* and NAC species. These results confirm the importance of the EGFR-MAPK signaling in VVC pathogenesis and highlight the remarkable immunogenic differences between *C. albicans* and NAC species in host-microbe interactions.

## Introduction

Vulvovaginal candidiasis (VVC) is the most common mucosal infection, characterized by symptomatic inflammation of the vagina (1). VVC has been reported to affect 70–75% of healthy women during their reproductive years, and about 5–8% of them evolve into recurrent VVC (RVVC) (2, 3). For *Candida* infection in the oral mucosa, both early innate immune responses and T cell-mediated adaptive immunity are critical and protective against the invasion of *Candida* pathogen (4–8). On the contrary, human challenge studies indicate that adaptive immune response is not defective and provides no protection upon *Candida* stimulation in both VVC and RVVC patients (9–11). Moreover, while an aggressive innate immune response is the primary determinant of the pathological process of VVC, fungal factors such as the presence of pseudo hyphae/hyphae are required but not sufficient to induce the pathological process of VVC (12). It seems that the hyperactive inflammatory response due to strong neutrophil recruitments and mucosal damage is responsible for symptoms of VVC (1). However, elevated neutrophil infiltration was demonstrated to be ineffective at reducing fungal burden and invasion during VVC (13–15), which appears to contradict the protective role of neutrophil recruitment in oral cavity or gastrointestinal tract for *Candida* clearance at the site of infection (6). Therefore, host mediated-epithelial damage and inflammatory response appear to be more important during the pathogenesis of VVC.

The mucosal epithelium not only functions as a physical barrier restraining commensal microbes, but also plays a critical role in orchestrating immune responses to activate myeloid cells in the submucosal layers to clear the invading pathogens (16, 17). The mechanism of how oral epithelial cells respond to candidal pathogens is well characterized (8, 18, 19) and epidermal growth factor receptor (EGFR) has also been shown to facilitate epithelial cell invasion *via* endocytosis of *C. albicans* while simultaneously promoting protective innate immune responses against *C. albicans (* 20, 21). EGFR is a membrane-bound tyrosine kinase receptor. After binding to the fungal ligands, EGFR signaling will activate inflammatory response *via* several pathways, including MAPK and nuclear factor kappa light chain enhancer of activated B cells (NF-ĸB) (20, 22, 23). MAP kinase phosphatase 1(MKP1) and c-Fos in the MAPK signaling pathway are both activated by the filamentous form of *C. albicans* in a biphasic manner in oral and vaginal epithelial infection *in vitro* (24, 25). However, the function of EGFR and its link to the main MAPK proteins (ERK1/2, JNK and p38) in VVC have never been explored.

The significance of pathogenic variation between different strains of *C. albicans* has been highlighted by a number of comparative studies. From the standpoint of fungus factors, genetic variation at the ECE1 locus, encoding a critical toxin of *C. albicans* (candidalysin), has recently been shown to influence the pathogenicity of *C. albicans* strains, SC5314 and 529L (26). Clinical *C. albicans* vaginal isolates and the standard strain SC5314 also differ during interaction with macrophages (27). By the same token, the immune responses of vaginal epithelial cells to clinical *C. albicans* strain and SC5314 could also be differently initiated in the context of VVC. Given the increasing population share of antifungal-resistant *Candida* spp. among clinical isolates over the past several decades (28), particular to azoles (29, 30), understanding how immune system responds to drug-resistant *Candida* also help us to develop more effective immunotherapeutic agents against drug-susceptible and-resistant fungi.

Despite the fact that *C. albicans* remains the principal etiologic agent of VVC, results from others also reveal an increasing prevalence of non-albicans *Candida* (NAC) species globally, affecting 10– 30% of VVC cases (31). Most of these cases are due to *C. glabrata*, followed by *C. parapsilosis*, *C. tropicalis*, *C. krusei* and *C. dubliniensis (* 32, 33). Compared with *C. albicans*, NAC species are generally more resistant to antifungal drugs (34), making treatment for VVC more complicated. Although less commonly reported, the highly antifungal-resistant emerging pathogen *C. auris* was described in one case of VVC (35). In a study with VVC murine model, although single isolate of each NAC species was tested, the preliminary data indicated that most NAC species are incapable of forming hyphae and do not elicit robust immunopathology *in vivo (* 36), but NAC do actively contribute to symptomatic VVC (37, 38). While significant attention has been given to *C. albicans*-induced host immune response, little is known regarding the interaction of vaginal epithelial cells and clinical NAC species.

To elucidate the function of EGFR and MAPK pathways in the pathogenesis of vulvovaginal candidiasis, we analyzed the immune activation of vaginal epithelial cells infected by both *C. albicans* and some NAC species with respect to the two signaling pathways. Our results reveal that EGFR is significantly activated by *C. albicans* in human vaginal epithelial cells both *in vitro* and *in vivo*, which governs the activation of MAPK proteins (p38 and ERK1/2). While the highly azole-resistant *C. albicans* induced stronger phosphorylation of EGFR and p38 than the drug-susceptible SC5314, the clinical NAC isolates and *C. auris* induced stronger phosphorylation of ERK1/2 and c-Fos in human vaginal epithelial cells than *C. albicans* strains. Furthermore, EGFR inhibitor greatly suppressed inflammatory cytokines production *in vitro* and also significantly decreased vaginal inflammation and fungal burden *in vivo* during *C. albicans* infection. Consistently, p38 and ERK1/2 inhibitor strongly suppressed inflammatory cytokines production but only the p38 inhibitor significantly improved cell survival of vaginal epithelium infected by both *C. albicans* and NAC species.

## Methods and Materials

### Culture of *Candida* Strains and Epithelial Cells

Clinical *C. albicans* isolates (1052 and C1-14) and NAC species (*Candida glabrata*, *Candida parapsilosis*, *Candida tropicalis*) were selected from our previous multicenter epidemiology study on VVC in China (38). Two *C. auris* isolates (10913 and 14918) were obtained from Wester Dijk Fungal Biodiversity Institute, and the standard strains (SC5314 and ATCC 90028) were reserved in the Institute of Dermatology, Chinese Academy of Medical Sciences. The susceptibility of two clinical *C. albicans* isolates (1052 and C1-14) to antifungal drugs was determined as described previously (39) and the minimum inhibitory concentration (MIC) values showed both isolates were highly azole-resistant (Fluconazole MIC >256). All cultures were streaked on yeast extract peptone dextrose (YPD) agar plates, and single colony was picked and grown in liquid YPD broth in an orbital shaker for 12h at 28°C (*C. albicans*, *C. tropicalis*, *C. glabrata* and *C. parapsilosis*) or 35°C (*C. auris*). Yeast cells were then pelleted by centrifugation and washed twice with PBS. The cells were suspended and counted *via* a hemacytometer. For Hyphae form of SC5314, the above yeast cells were grown in fresh 1640 medium and shaking at 37°C for 3-5 h.

The human vaginal epithelial cell line VK2/E6E7 cells (ATCC #CRL-2616, VA) were cultured in keratinocyte-serum free medium (KSFM) (Gibco, USA) supplemented with 50 μg/ml of bovine pituitary extract (BPE) and 0.1 ng/ml of epidermal growth factor (EGF) at 37°C with 5% CO_2_. Experiments were carried out in KSFM without supplements and performed independently at least 3 times.

### RNA Sequencing

After 6h stimulation with *C. albicans*, VK2/E6E7 cells were rinsed with cold PBS and the total RNA was extracted using Tri reagent solution (Ambion). RNA-seq libraries were constructed according to the technical protocol and single-end 50-base reads were produced on a BGISEQ-500 platform at BGI-Shenzhen following the manufacturer’s procedures. Genes were considered significantly differentially expressed according to the criteria of a fold change ≥2 and Q value (adjusted P value) ≤0.05.

### Immunoblot of Protein *In Vitro* and *In Vivo*

The VK2/E6E7 vaginal epithelial cells were seeded onto six-well tissue culture plates and incubated in supplement-free KSFM for 12 h and then infected with *Candida* cells with a multiplicity of infection (MOI) of 5. EGFR inhibitors were added to the host cells 2h before the fungal stimulation. At various time points, the epithelial cells were rinsed with cold PBS and lysed using a modified RIPA lysis buffer containing protease (Cell Signaling Technology) and phosphatase (Sigma-Aldrich) inhibitors, left on ice for 30 min. The cells were collected by centrifugation and supernatants were assayed for total protein. 20 ug of the protein was separated by SDS-PAGE and the proteins were detected by immunoblotting with specific antibodies, including anti-phospho-EGFR Tyr1068 (#3777), anti-phospho-c-Fos Ser32 (Cell signaling; #5348), anti-phospho-p65 Ser536 (Cell signaling; #3033), anti-phospho-JNK Thr183/Tyr185 (Cell signaling; #9255),anti-phospho-Erk1/2 Thr202/Thr204 (Cell signaling; #4370), anti-phospho-p38 Thr180/Tyr182 (Cell signaling; #4511). For the extraction of total protein from vaginal tissue, half of the dissected vagina was firstly grinded and then lysed using Minute™ Total Protein Extraction Kit for Animal Cultured Cells/Tissues (SD-001/SN-002, invent biotechnologies, America) at 4°C. After quantitation, the tissue protein was separated by SDS-PAGE and detected as above.

### Overexpression of EGFR

VK2/E6E7 cells were plated into 6-well tissue culture plates at 6.0 × 10^5^ cells per well and then transfected with the lentivirus (Hanbio Biotechnology, Shanghai, China) containing LV-EGFR-sh2 for 24h according to the manufacturer’s protocol. The overexpression efficiencies of EGFR were identified using Western blotting.

### Epithelial Cell Damage in Real-Time Cell Analysis Experiments

The xCELLigence Real-Time Cell Analyzer (RTCA) system (Acea Biosciences, San Diego, CA, USA) was adopted to measure cell damage induced by *Candida* pathogens. VK2/E6E7 cells were counted and then seeded (5×10^4^) into the E-plate 96 wells, incubating at 37°C for at least 20 h prior to treatment. When the cell growth curve reached a plateau, inhibitors or DMSO (0.8%) were added to the host cells 2h prior to the fungal stimulation and the vaginal epithelial cells were infected by different *Candida* strains at MOI of 10. EGFR inhibitor AG1478(Absin, abs810610) was used at 4.5μM, ERK1/2 inhibitor SD5978(Biyotime) was used at 5μM and p38 inhibitor SB203580 (selleckchem) was used at 5uM. The cell index was automatically recorded every 15 min during the incubation period. This experiment was performed in duplicates and run for 24h postinfection.

### Cytokine Measurements *In Vitro*

The VK2/E6E7 cells were grown in 6-well tissue culture plates for 48h and then infected with *Candida* yeast cells at MOI of 0.01 for 24 h. The culture supernatants were collected and clarified by centrifugation. The levels of CCL20, GM-CSF, G-CSF, IL-1β, IL-6 and IL-17A in the supernatants were determined using the Luminex multipex assay (R&D Systems; number LXSAHM-08).

### Murine Model of VVC

Our previously described murine model of vulvovaginal candidiasis was modified for investigating early vaginal infection events (40). The estrogen (β-estradiol 17-valerate; Sigma) was firstly dissolved in DMSO (20mg/mL) and then in sesame oil (2mg/mL). The female mice were injected subcutaneously with 0.2 mg of estrogen every other day to a total of three times. The mice were then anesthetized with 1 mg/kg urethane and were inoculated into the vaginal lumen with 10 µL of 2×108 cfu/ml *C. albicans* yeast in sterile saline. After 1 day, mice were sacrificed, the vagina was harvested and divided by two. One-half was weighed and homogenized for Immunoblotting. The other one-half was processed for histopathology and immunofluorescence. For EGFR inhibition studies, the mice were administrated with 30 mg/kg AG1478 at day -2 and day -1 with respect to *C. albicans* inoculation. Mice were infected with *C. albicans* spores and processed as above.

### Histopathology and Immunofluorescence of Vaginal Tissues

The vaginal tissue was transferred to 4% paraformaldehyde solution, embedded in paraffin and sectioned at an optimal cutting temperature. 5-µm sections were selected and stained with Periodic Acid-Schiff (PAS), hematoxylin-eosin (H&E) or antibody. Stained sections were scanned and evaluated for severity of inflammation and fungal burden. For immunofluorescence, slides were probed with indicated antibodies at 4°C overnight and washed in PBS three times the next day, followed by fluorescence-conjugated secondary antibodies. They were subjected to DAPI counter-staining before being air-dried and mounted. Immunofluorescent images were collected by LSM 510 Laser Scanning Microscope and analyzed by ImageJ software.

### Statistics

Continuous variables are expressed as the means ± standard deviation(SD), and categorical data are presented as frequencies or percentages. we performed the student’s t-test (comparing the two groups) and one-way ANOVA (comparing three or more groups), followed by Tukey’s (comparing all groups) or Dunnett’s (comparing treatments vs. control) post-tests to assess the significance of changes relative to controls. 2-sided tests were used in all statistical analyses and a P value<0.05 was considered significant. All tests were performed with the GraphPad Prism 9.0.

## Results

### Activated EGFR Governs the Activation of MAPK Pathway in Vaginal Epithelial Cells by *C. albicans In Vitro* and *In Vivo*

**Figure 1:**
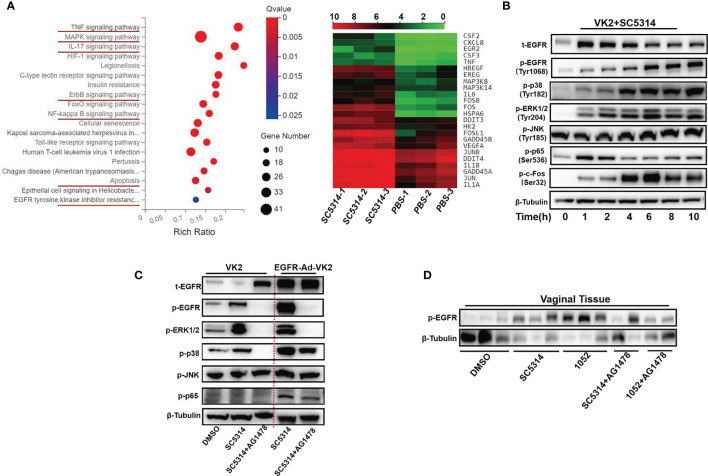
*C. albicans* activates EGFR, NF-κB, MAPK and c-Fos signaling in vaginal epithelial cells. Pathway enrichment analysis and heatmaps of VK2/E6E7 vaginal epithelial cells stimulating with SC5314 and PBS. Using the phyper function in the R software to perform enrichment analysis and calculate the P value. Then performing FDR correction on the P value to obtain the Q value. A Q value < 0.05 is regarded as a significant enrichment. Red underlines highlight the upregulated pathways related to EGFR and MAPK. Heat map displaying the upregulated(red) or downregulated(blue) genes. Each column represents an individual sample(n=3) **(A)**. VK2/E6E7 cells were stimulated with SC5314 (MOI=5) for the indicated time. Cell lysates were analyzed by immunoblotting for the indicated proteins. The phosphorylated forms of proteins are preceded by “p” **(B)**. SC5314 elicited significantly stronger phosphorylation of p38 in EGFR-Ad-VK2, VK2/E6E7 cells overexpressing EGFR, when compared to VK2/E6E7 cells. EGFR inhibitor (AG1478) suppressed SC5314-induced phosphorylation of ERK1/2 and p38 in EGFR-Ad-VK2 and VK2/E6E7 cells **(C)**. Female ICR mice (n=3 or 2/each group) were infected with SC5314 or clinical isolate1052 after pre-treatment with DMSO or AG1478 for 2d and vaginal tissues were harvested 1 d after infection for detection of EGFR phosphorylation by immunoblotting analysis **(D)**. Data are representative of three independent experiments and bands are shown relative to β-tubulin loading control.

To determine whether EGFR and possible downstream signaling pathways in vaginal epithelial cells are involved in VVC inflammation, we first performed RNA–seq analysis on VK2/E6E7 cells at 6 h post incubation with *C. albicans* standard strain SC5314. We found that ErbB, MAPK, NF-κB and IL-17 signaling pathways were significantly more up-regulated in stimulated VK2/E6E7 cells than phosphate-buffered saline (PBS)-treated cells (**Figure 1A**). The heat map shows most up-regulated genes including CSF2, CSF3, FOS, IL-1, GADD45 (**Figure 1A**). Since numerous studies have shown that phosphorylation of EGFR and MAPK signaling are critical for the activation of oral epithelial cells in response to *C. albicans* (20, 25, 41, 42), we next investigated the phosphorylation of EGFR, MAPK (p38, ERK1/2, JNK), c-Fos and NF-κB(p65) in VK2/E6E7 vaginal epithelial cells at different time points post co-culture with SC5314 at 5 of multiplicity of infection (MOI). The phosphorylation level of EGFR on tyrosine residue 1068 immediately increased after1 h post infection and sharply increased at 4-6 h and then remained high levels until 10 h post infection (**Figure 1B**). The trend of phosphorylated p38 elevations was similar to phosphorylated EGFR. The phosphorylation of ERK1/2 or c-Fos was also induced by the pathogen at 1 h post-infection and sustained until 10 h. However, phosphorylated ERK1/2 gradually reached its highest point at 6 h, accompanied with a sharp increase of phosphorylated c-Fos at 4 h (2 h earlier than p38) (**Figure 1B**). In conjunction with the different trajectory of these MAPKs, the phosphorylation of NF-κB quickly elevated during the first 2 h and subsided afterwards and phosphorylated JNK remained unchanged at each time points (**Figure 1B**). In terms of an earlier NF-κB response, our data suggest that EGFR signaling and its downstream MAPKs (p38, ERK1/2) may be more critical for *Candida*- induced earlier inflammatory response in vaginal epithelial cells. Among these MAPK(s), the time course of ERK1/2 activation is better lined up with c-Fos phosphorylation. Unchanged phosphorylation of JNK, opposite the responses of oral epithelial cells to the same *C. albicans* strain (25), argues roles of JNK in vaginal immune responses to *Candida* spp.

In oral epithelial cells, EGFR inhibitor can significantly suppress *C. albicans*-induced pEGFR, c-Fos and pMKP1 (20). To verify the EGFR roles in activating downstream target MAPK proteins, phosphorylated levels of three MAPKs (p38, ERK1/2, JNK) in *C. albicans* infected vaginal epithelial cells were assessed using an EGFR antagonist AG1478 and EGFR overexpressed epithelial cells (EGFR-Ad-VK2). We first treated VK2/E6E7 epithelial cells with AG1478 2 h prior to *C. albicans* challenge. As expected, the phosphorylations of EGFR, p38 and ERK1/2 at 6 h post-infection were completely inhibited by AG1478, while phosphorylated JNK remained the same level (**Figure 1C**). By contrast, *Candida*-induced EGFR and p38 phosphorylation levels, but not ERK1/2 phosphorylation, were increased in EGFR-Ad-VK2 cells that again were effectively reversed in the presence of AG1478 (**Figure 1C**). EGFR, p38 and ERK1/2 inhibitors did not affect *C. albicans* hyphal growth (**Figure S1**).

To verify EGFR signaling responses *in vivo*, we used a mouse VVC model to analyze activation of EGFR during VVC. We found that infections with standard *C. albicans* SC5314 and drug resistant strain 1052 can induce high level of phosphorylated EGFR in collected vaginal tissues, especially in the case of 1052 infection (**Figure 1D**). Treatment of mice with EGFR antagonist AG1478 greatly reduced phosphorylation of EGFR in both C. albicans infected mice, especially in 1052 infected vaginal tissues (**Figure 1D**). Taken together, *in vivo* results agree with *in vitro* data to show that EGFR signaling is necessary for activating *C. albicans*-induced vaginal inflammation.

### The Role of EGFR/p38 (ERK1/2) Pathway in Mediating *C. albicans* Induced Inflammatory Responses in Vaginal Epithelial Cells

**Figure 2:**
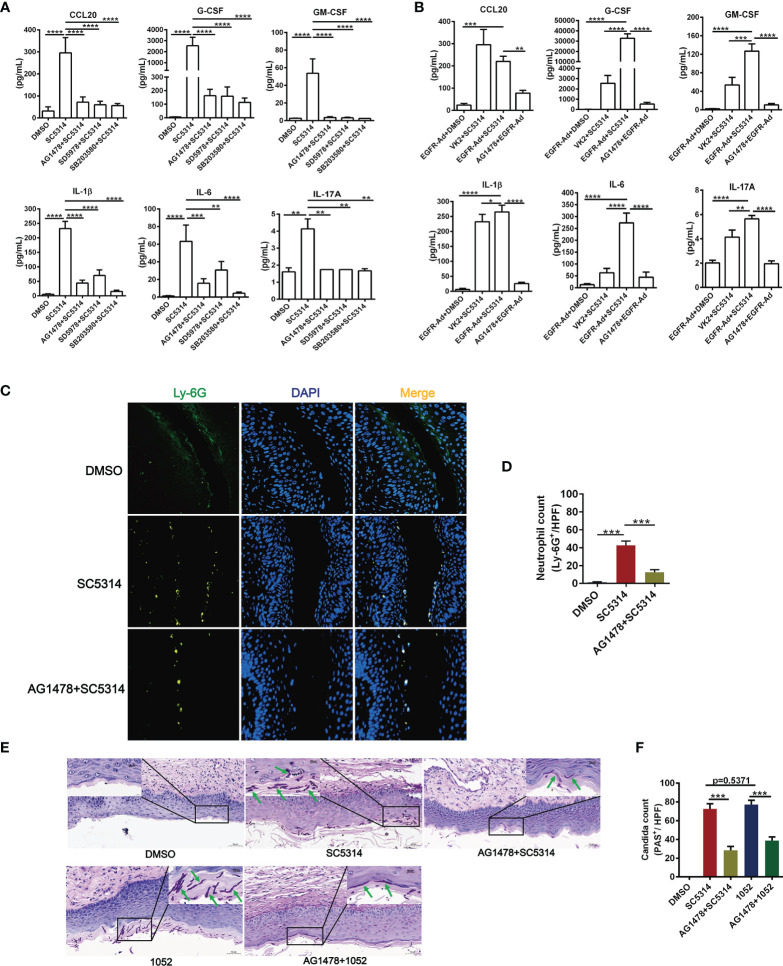
Functional role of EGFR and MAPK signaling in regulating vaginal epithelial inflammatory responses. Quantification of cytokines were assessed by multiplex microbead assay (luminex), secreted from VK2/E6E7 vaginal epithelial cells stimulating with SC5314(MOI=0.01) for 24 h. EGFR inhibitor AG1478, p38 inhibitor SB203580 or ERK1/2 inhibitor SD5978 was added to the cells 2 h prior to fungal stimulation. All these inhibitors suppressed SC5314-induced secretion of cytokines (CCL20, G-CSF, GM-CSF, IL-1β, IL-6, IL-17A) **(A)**. EGFR-Ad-VK2 released higher levels of cytokines (G-CSF, GM-CSF, IL-1β, IL-6, IL-17A) induced by SC5314 when compared to VK2/E6E7 cells, which can be suppressed by AG1478 **(B)**. VVC mice were treated as previously (**Figure 1D**). Vaginal sections from *C. albicans*–infected mice were assayed for immunofluorescence staining of Ly-6G (neutrophil marker, green) and nuclei were counterstained with DAPI (blue) **(C)**. Numbers in graph **(D)** provide the mean neutrophils count per group (n=3/each group). Fungal load in vaginal tissues was assessed by periodic acid-Schiff (PAS). Insets show regions of vaginal fungal burden and higher-magnification images of the organisms indicated by the arrows **(E)**. Numbers in graph **(F)** provide the mean *C. albicans* count per group (n=3/each group). Data are representative of three independent experiments. Error bars represent SD, *p < 0.05, **p < 0.01, ***p < 0.001, ****p < 0.0001.

The *in vitro* data above suggest that EGFR signaling *via* p38 and ERK1/2 pathway activates *C. albicans*-induced inflammatory response in vaginal epithelial cells. To understand the impact of EGFR signaling activation for the downstream inflammatory response during vaginal inflammatory response, antagonists AG1478 (EGFR), SD5978 (ERK1/2) and SB203580 (p38) were used to treat VK2/E6E7 vaginal epithelial cells 2 h prior to infection with SC5314. We found that inhibition of phosphorylation of EGFR, ERK1/2 or p38 respectively resulted in significant reduction in the levels of CCL20 (75.70%, 79.73%, 80.94%), G-CSF (93.61%, 93.75%, 95.53%), GM-CSF (93.33%, 94.15%, 95.38%), IL-1β (81.04%, 69.96%, 93.46%), IL-6 (75.32%, 51.58%, 93.26%) and IL-17A (57.94%, 57.94%, 59.63%) (**Figure 2A**). Among the three inhibitors, SB203580 showed the strongest inhibitory effect on all the cytokines induced by SC5314, particularly on G-CSF, IL-1β and IL-6.

Compared with SC5314-infected VK2/E6E7 cells, EGFR-Ad-VK2 cells secreted much higher levels of cytokines such as G-CSF, GM-CSF, IL-1β, IL-6 and IL-17A – except CCL20 upon SC5314 stimulation (**Figure 2B**). Although the CCL20 level in SC5314-infected EGFR-Ad-VK2 was more highly produced than in the DMSO control, the quantity was less than for SC5314 infected VK2/E6E7 cells. Again, the upregulation of all the cytokines – including CCL20 in EGFR-Ad-VK2 cells – can be suppressed by EGFR, p38 and ERK1/2 inhibitors as effectively as those in infected VK2/E6E7 cells (**Figure 2B**). Moreover, in the murine model of VVC, treatment of AG1478 greatly ameliorated vaginal inflammation caused by SC5314. The effects of AG1478 were confirmed by histopathologic and immunofluorescent analysis, which revealed that the vaginas of AG1478-treated mice had low levels of neutrophil recruitment (**Figures 2C, D**) and fungal burden (**Figures 2E, F**). In all, these data imply that EGFR-p38/ERK1/2 pathways are required for vaginal epithelial cells to mount inflammatory responses to *C. albicans* and inhibition of EGFR signaling would reduce vaginal neutrophil recruitment and fungal burden during VVC.

### High Activation of the EGFR Pathway in Vaginal Epithelial Cells by Clinical Drug-Resistant *C. albicans* Isolates

**Figure 3:**
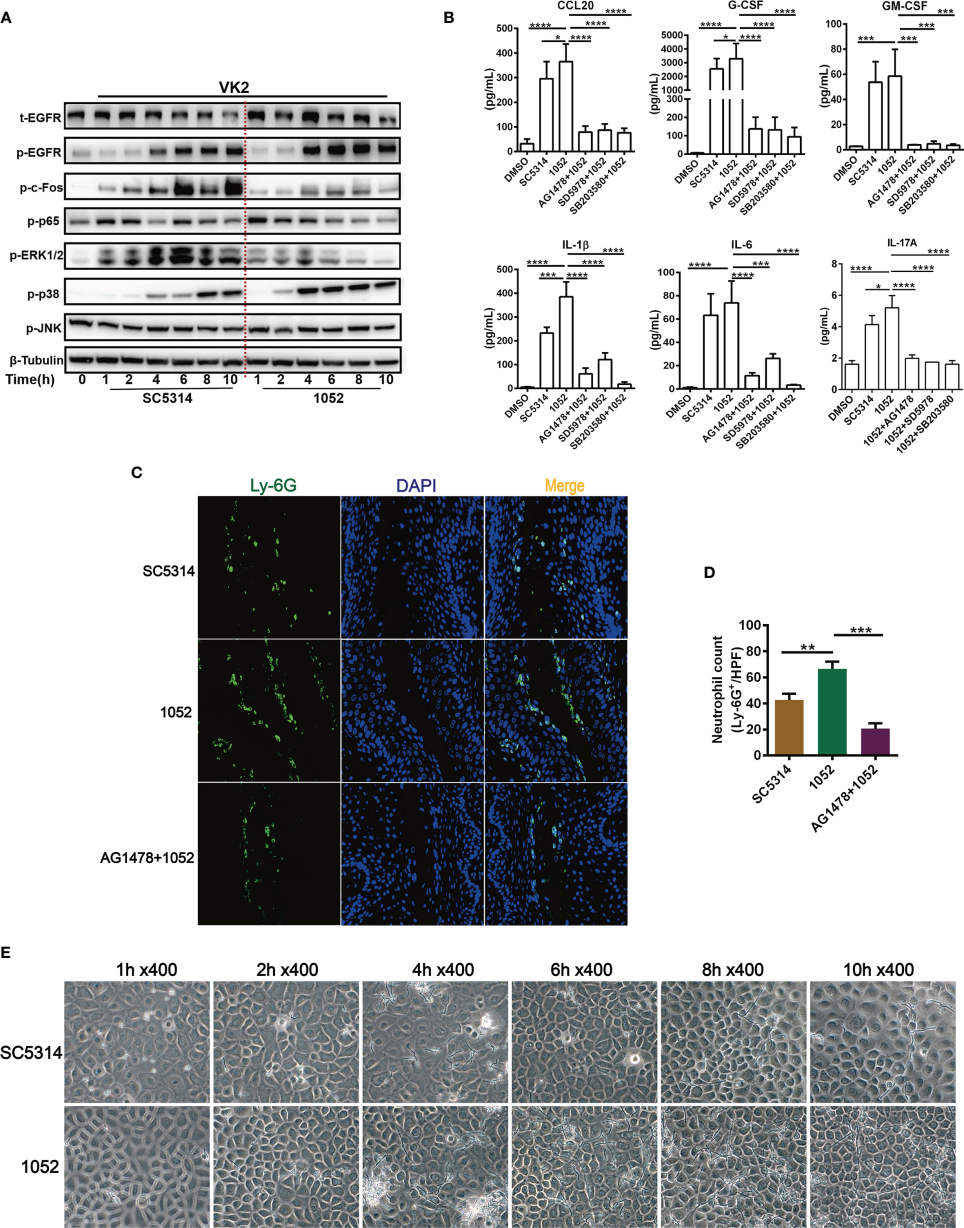
Differential activation of EGFR and MAPK and cytokine induction by clinical isolate 1052 and WT strain SC5314. VK2/E6E7 cells were stimulated with SC5314 and clinical isolate 1052 (MOI=5) for the indicated time respectively. Phosphorylation of EGFR, MAPK proteins, NF-κB (p65) and c-Fos was detected by immunoblotting of cell lysates **(A)**. 1052 induced higher levels of cytokines secretion (CCL20, G-CSF, GM-CSF, IL-1β, IL-6, IL-17A) than SC5314. All inhibitors (EGFR, p38 and ERK1/2 inhibitor) suppressed 1052-induced secretion of CCL20, G-CSF, GM-CSF, IL-1β, IL-6 and IL-17A **(B)**. Infection with 1052 in the vaginal mucosa of mice induced significantly increased neutrophil recruitment at the infection foci when compared with SC5314 and pre-treatment of AG1478 resulted in significantly decreased neutrophil recruitment **(C)**. Numbers in graph **(D)** provide the mean neutrophils count per group (n=3/each group). Morphology of SC5314 and clinical isolate 1052 adhering to VK2/E6E7 cells at 0, 1, 2, 4, 6, 8, 10 h post infection **(E)**, gently washed with PBS for at least 3 times. Data are representative of three independent experiments. Error bars represent SD, *p < 0.05, **p < 0.01, ***p < 0.001, ****p < 0.0001.

The above experiments were mostly performed with the standard *C. albicans* strain SC5314, an original clinical isolate that has been widely used to evaluate fungal and host interaction. Pathogenic variability has been noted in *C. albicans* isolates (6). To discriminate the possible difference in host responses to clinical drug-sensitive and drug-resistant *C. albicans*, the EGFR responses in VK2/E6E7 cells infected by SC5314 (drug-sensitive) and a clinical azole-resistant *C. albicans* 1052 (38) were compared. Total proteins of VK2/E6E7 cells were extracted at 1, 2, 4, 6, 8 and 10 h post fungal infection for measurement of the activation of EGFR-MAPK pathway. We found that EGFR and p38 phosphorylation were significantly higher in 1052 infected epithelial cells, especially in the 4 to 10 h period (**Figure 3A**). We find it intriguing that ERK1/2 phosphorylation induced by 1052 appeared at early stages (1- 4 h) and gradually subsided at later stages, which was different from an overall increased ERK1/2 response in SC5314 infection especially (**Figure 3A**). In accord with the early ERK1/2 response in both strain infections, p65(NF-κB) phosphorylation levels were higher in SC5314 and 1052 infected epithelial cells during 1- 4 h. The second difference between two types of infections was that the levels of c-Fos phosphorylation induced by 1052 were significantly lower than the levels induced by SC5314 at both early and later stage (**Figure 3A**).

To better understand the meanings of these divergent host responses in fungal infection, we collected micrographs of *Candida* morphology adhering to vaginal epithelial cells at different time points. We found that the azole-resistant isolate was more filamentous than SC5314. The hyphal lengths of 1052 adhering to VK2/E6E7 cells were longer and the quantities of hyphae were greater than SC5314 at each time point after 2 h (**Figure 3E**). In accord with these *in vitro* results, the hyphal lengths of 1052 adhering to vaginal mucosa were also longer than SC5314 in the mouse model of VVC at 24 h post infection (**Figure 2E**). The different MAPK responses in vaginas infected by drug-sensitive and drug-resistant *C. albicans* certainly require a further investigation with more strains. However, the extent of hyphal formation could be a convenient explanation for such a difference in MAPK responses. Nevertheless, EGFR responses are better lined up with p38 response in both strain infections.

The inflammatory cytokines in supernatants of VK2/E6E7 cells infected by SC5314 and 1052 were also measured and compared. The results showed that levels of all cytokines (CCL20, G-CSF, GM-CSF, IL-1β, IL-6 and IL-17A) in 1052 infected epithelial cells were higher than in SC5314 infected cells and each highly-produced cytokine in the 1052-stimulated epithelial cells can be suppressed by EGFR, p38 and ERK1/2 inhibitors (**Figure 3B**). In particular, the inhibition of p38 resulted in a much greater reduction of 1052-triggered G-CSF, IL-1β and IL-6 than the other two inhibitors (**Figure 3B**). Along with stronger inflammatory cytokine production, levels of neutrophil infiltrates in the vaginal tissue infected by 1052 were significantly higher than SC5314 (**Figures 3C, D**). Apparently, inflammatory cytokines and neutrophil infiltrate levels were dependent on EGFR since both phenotypes in SC5314 and 1052 infection can be reversed by AG1478(**Figures 3C, D**).

### Differential Activation of the EGFR Pathway in Vaginal Epithelial Cells by *C. albicans* and Non-Albicans *Candida* Species

**Figure 4:**
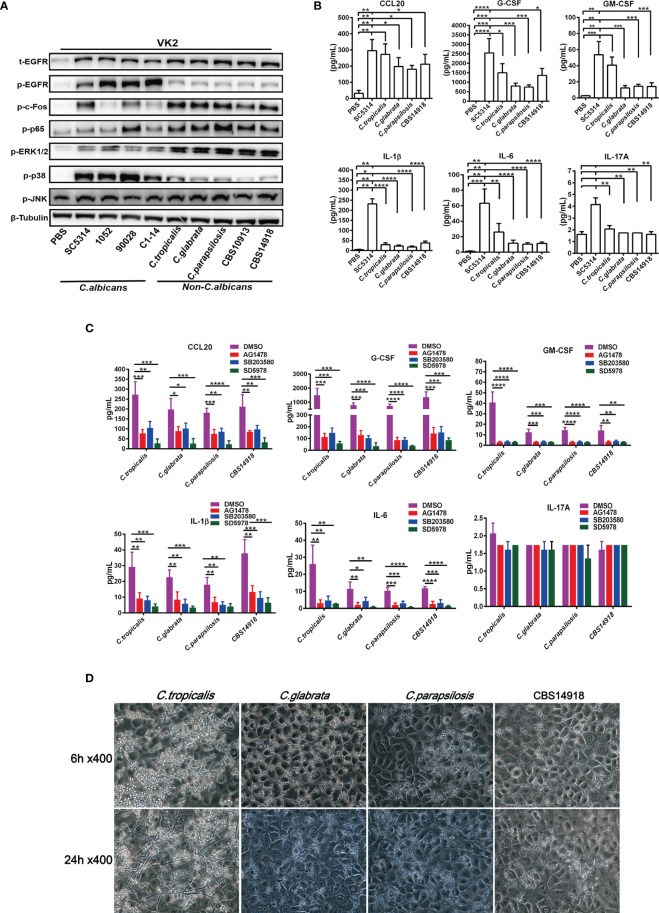
Divergent activation of EGFR and MAPK and cytokine induction by *C. albicans* and non-albicans *Candida* species. VK2/E6E7 cells were stimulated with *C. albicans* (SC5314, 1052, ATCC90028 and C1-14) and non-albicans *Candida* (NAC) isolates *C. tropicalis*, *C. glabrata*, *C. parapsilosis*, CBS10913 and CBS14918) (MOI=5) respectively. Protein lysates were taken at 6 h post-infection for western blotting analysis **(A)**. Cytokines induced by *C. albicans* and NAC isolates (MOI=0.01) were assessed at 24h post-infection *via* Luminex **(B)**. All inhibitors suppressed NAC isolates-induced cytokines production **(C)**. Morphology of *C. tropicalis*, *C. glabrata*, *C. parapsilosis* and CBS14918 strains on VK2/E6E7 cells at 6 and 24 h post infection **(D)**. Data are representative of three independent experiments. Error bars represent SD, *p < 0.05, **p < 0.01, ***p < 0.001, ****p < 0.0001.

Vulvovaginal candidiasis (VVC) caused by non-albicans *Candida* (NAC) species are increasingly reported today. Given that more drug-resistant strains have been found in NAC species, we next analyze the EGFR-MAPK responses during the NAC species infections by using 3 clinical NAC isolates of *C. tropicalis*, *C. glabrata*, *C. parapsilosis* previously isolated from VVC patients (38) and 2 strains of *C. auris* (CBS10913 and CBS14918). The results were compared with 4 strains of *C. albicans* (SC5314, ATCC90028, 1052 and C1-14). To examine the extent of EGFR/MAPK pathway activation in vaginal epithelial cells infected by each of these strains, proteins of infected epithelial cells 6 h post infection at an MOI of 5 were extracted for analysis. We found that 4 C*. albicans* strains induced significantly higher levels of EGFR and p38 phosphorylation in VK2/E6E7 cells than all NAC strains (**Figure 4A**). When compared with the *C. albicans* strains, NAC strains consistently triggered much stronger activation of ERK1/2 and c-Fos phosphorylation in VK2/E6E7 cells (**Figure 4A**). In general, there were no significant difference in phosphorylation of EGFR, p38 or ERK1/2 among these NAC infected epithelial cells except that a slightly decreased ERK1/2 phosphorylation and a slightly increased p38 phosphorylation were seen in *C. tropicalis* (**Figure 4A**). When one consideres that *C. tropicalis* is phylogenically closer to *C. albicans* than other species, the ERK1/2 and p38 responses observed in *C. tropicalis* are not surprising. Nevertheless, activation of ERK1/2 and c-Fos phosphorylation seems to mediate the immune responses of vaginal epithelial cells by NAC species.

Typically, MAPK signaling in oral epithelial cells is correlated with hyphal formation of *C. albicans (* 41). However, clinical NAC species used in this study that were not able to form the true hyphae in epithelial cells (**Figure 4D**) also effectively activated ERK1/2 and the protein c-Fos (**Figure 4A**). We found that only a small population of *C. tropicalis* switched to pseudohyphae, while *C. glabrata*, *C. parapsiolosis* and *C. auris* grew only in the yeast morphology until 24 h (**Figure 4D**). These results suggested that ERK1/2 phosphorylation may be more yeast-specific while EGFR and p38 phosphorylation are more hyphae-specific in vaginal epithelial cells, which is also supported by the higher EGFR and p38 responses induced by drug resistant 1052 with high filamentation in **Figures 3A, E**.

Regarding the downstream cytokine levels, 4 NAC species generally triggered less cytokine production in VK2/E6E7 cells when compared to SC5314 (**Figure 4B**). Inhibition of EGFR, p38 and ERK1/2 led to further reductions of all cytokines by 4 NAC species (**Figure 4C**) except IL-17A. Particularly, ERK1/2 inhibitor showed the strongest inhibitory effects on NAC-induced CCL20, G-CSF, GM-CSF, IL-1β, and IL-6, which is consistent with the strong activation of ERK1/2 phosphorylation induced by NAC species (**Figure 4A**). This highlights the importance of ERK1/2 in mediating the inflammatory responses in NAC species-induced vaginal infection.

### Function of EGFR/MAPK Pathway in Moderating Epithelial Damage During VVC

**Figure 5:**
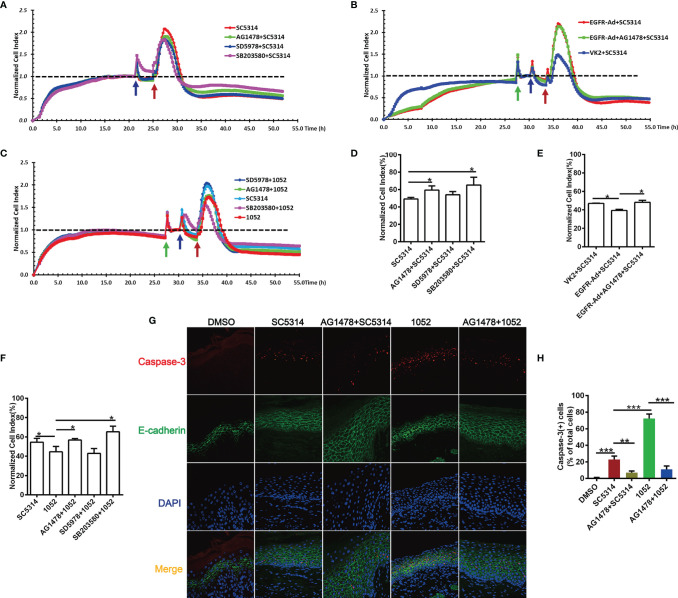
EGFR and MAPK signaling regulate the viability of vaginal epithelial cells after infection with *C. albicans.* Cell damage was monitored continuously by xCELLigence Real-Time Cellular Analysis (RTCA) system. Normalized Cell Index (NCI) demonstrated cell viability. 1052-infected VK2/E6E7 cells exhibited a significant decrease in viability when compared to SC5314 infections and pre-treatment of the EGFR and p38 inhibitors protected cells against SC5314 or 1052-killing **(A, C)**. SC5314-infected EGFR-Ad cells exhibited increased damage when compared to the infected VK2/E6E7 cells **(B)**. NCI at 24 h post infection in graph **(D–F)** provides the mean cell viability. Green arrow represents the time points on which the medium was changed. Blue and red arrows represent the time point at which inhibitors and *Candida* strains were added into the well respectively. VVC mice were treated as previously (**Figure 1D**). Immunofluorescent staining of E-cadherin (green), caspase-3(red) and DAPI (blue) demonstrated apoptotic vaginal epithelial cells in vaginal tissues sections **(G)**. Numbers in graph **(H)** provide the mean apoptotic vaginal epithelial cells count per group (n=3/each group). Data are representative of three independent experiments. Error bars represent SD, *p < 0.05, Error bars represent SD, *p < 0.05, **p < 0.01, ***p < 0.001.

In addition to vaginal inflammation, epithelial damage also contributes to the symptoms of VVC (1, 43). Since inhibition of EGFR and p38/ERK1/2 can effectively reduce epithelial inflammatory responses to SC5314, we then wonder whether blocking these pathways would lessen epithelial damage during the VVC infection. The real-time damage of vaginal epithelial cells during *Candida* infection was monitored using an xCELLigence (real time cell analyzer) RTCA Instrument. For SC5314 infection, normalized cell index of VK2/E6E7 cells increased sharply in the first 2 hours and then subsided. However, EGFR and p38 inhibitors, but not ERK1/2 inhibitor, significantly improved cell survival of the infected VK2/E6E7 cells at 24 h post infection (**Figures 5A, D**). Conversely, EGFR-Ad-VK2 cells resulted in a significant increase in cell death when infected by SC5314 (**Figures 5B, E**). EGFR inhibitor *in vivo* also greatly reduced the apoptosis of vaginal epithelium (**Figures 5G, H**). Taken together, these data clearly demonstrated that the EGFR/p38 pathway is critical in driving the epithelial damage caused by *C. albicans* during VVC.

Considering the fact that 1052 activated higher levels of EGFR and p38 phosphorylation, we hypothesized that the azole-resistant isolate may cause more damage to vaginal epithelial cells than SC5314. As expected, the viability of VK2/E6E7 cells stimulated with 1052 was reduced compared to the viability of SC5314-infected cells (**Figures 5C, F**). Similarly, blocking EGFR and p38 resulted in a significant increase in cell survival, but ERK1/2 inhibitor had no effect on cell viability (**Figures 5C, F**). In the murine model of VVC, levels of cell apoptosis in 1052-infected vaginal tissue were significantly higher than in SC5314-infected vaginal tissue and blocking EGFR also decreased cell apoptosis induced by 1052 (**Figures 5G, H**).

**Figure 6:**
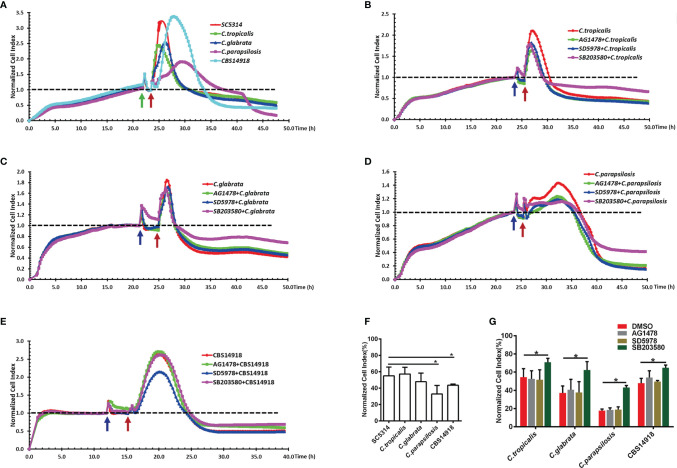
EGFR and MAPK signaling regulate the viability of vaginal epithelial cells after infection with non-albicans *Candida* species. Cells damage caused by SC5314 and non-albicans *Candida* (NAC) isolates were monitored continuously for 24 h **(A)**. Only pre-treatment of p38 inhibitor (SB203580) exhibited a significant increased protective capacity against NAC isolates compared to EGFR and ERK1/2 inhibitor **(B–E)**. NCI at 24 h post infection in graph **(F, G)** provides the mean cell viability. Green arrow represents the time points at which the medium was changed. Blue and red arrows represent the time point on which inhibitors and *Candida* strains were added into the well respectively. Data are representative of three independent experiments. Error bars represent SD, *p < 0.05.

Despite lower levels of phosphorylation of EGFR and p38 in NAC infections than *C. albicans* infection (**Figure 4A**), epithelial damage caused by *C. tropicalis* and *C. glabrata* was as great as that caused by *C. albicans*; cell damage by *C. parapsilosis* and *C. auris* was even more extensive than *C. albicans* (**Figures 6A, F**). For all the NAS strains, the p38 inhibitor significantly improved the epithelial cell survival during the infection, but this was not the case for the EGFR or ERK1/2 inhibitor (**Figures 6B–E, G**), which highlights that p38 signaling regulates NAC-induced epithelial damage. While both EGFR and p38 are required for *C. albicans*-induced epithelial damage (**Figure 5D**), the absence of EGFR response in NAC induced epithelial damage (**Figure 6G**) suggests a likely different upstream signal for p38-mediated epithelial responses in NAC infections.

## Discussion

**Figure 7:**
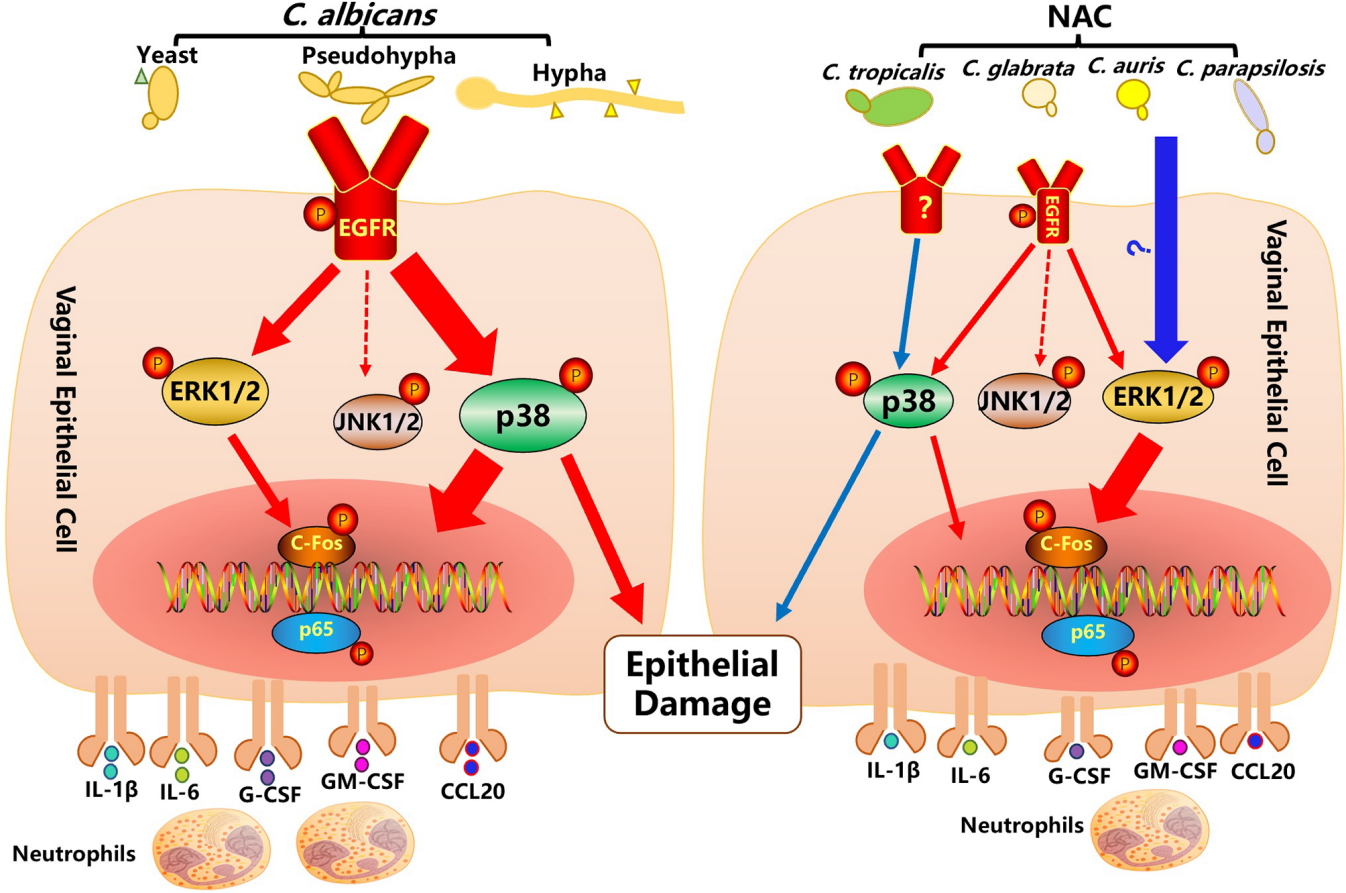
Activation of the EGFR-MAPK signaling pathway in vaginal epithelial cells by *Candida* pathogens. *C. albicans* infection on vaginal epithelial cells manifests by a modest yeast-to-hyphal transition within the first 2 hours, when the EGFR-ERK1/2 signaling pathway (small red arrow) is moderately activated. At a later stage of stimulation (4 to 10 hours), after the formation of a greater number of hyphae, the EGFR-p38 signaling pathway (thick red arrow) is strongly activated. Activated EGFR-p38 and-ERK1/2 signaling pathways lead to an increased phosphorylation of c-Fos and p65 and an increased secretion of cytokines (IL-1, IL-6, G-CSF, GM-CSF, CCL20). In particular, the EGFR-p38 signaling pathway also moderates epithelial damage induced by *C. albicans*. While non-albicans *Candida* (NAC) spp. infections of vaginal epithelial cells rarely produce hyphae, they activate a strong phosphorylation of ERK1/2, c-fos and p65 and a subsequent secretion of cytokines. Although not strongly activated, the p38 pathway still has some effect in the moderation of epithelial damage.

The pathological process of VVC is largely driven by local innate immune response, where vaginal epithelial cells have been shown to play a central role (1, 12). In this study, the function of the EGFR signaling pathway and the possible key players are investigated in mouse VVC infected tissue and *Candida* stimulated vaginal epithelial cells *in vitro*. Through divergent patterns of EGFR and respective downstream MAPK proteins activated by *C. albicans*, drug resistant *C. albicans* and non-albicans *Candida* species, our *in vivo* and *in vitro* data confirm that the EGFR/MAPK pathway in vaginal epithelial cells is sustained by *Candida* stimulation with a time course that is different from the biphasic manner of oral epithelial cells based on yeast to hyphal conversion (17). We see that *C. albicans* prefers to induce an EGFR-p38 mediated inflammatory response and epithelial damage, while NAC species elevate phosphorylated levels of ERK1/2 and c-Fos for downstream NK-ĸB activation and cytokine productions, however, p38 is still required for the promotion of epithelial damage (summarized in **Figure 7**).

In *Candida*-infected oral epithelial cells, activation of EGFR and downstream MAPK target proteins (c-Fos and MKP1) are key for candidalysin-induced immune responses (20, 44). Activation of this c-Fos/MKP1 signaling is also associated with a biphasic immune response pattern that enable oral epithelial cells to discriminate the morphology of *C. albicans* (25). During this biphasic response, phosphorylation of p38 and JNK induced by *C. albicans* reach their highest levels at 2 h post-infection and then decrease in oral epithelial cells. However, in *Candida*-infected vaginal epithelial cells in this study, phosphorylation of p38 increased over time and peaked at a later stage. In addition, phosphorylated JNK remains normal during at the time points. Our data support the notion that the variations in mucosal responses against *C. albicans* are niche-specific (6). As the over expression of EGFR in VK2/E6E7 cells resulted in a significant increase of p38 phosphorylation, but not of ERK1/2 phosphorylation, we believe that the primary downstream target protein of an activating EGFR signaling for inflammatory cytokines during VVC infection is p38 MAPK.

Symptomatic VVC is associated with massive neutrophil migration to the vagina and the subsequent uncontrolled inflammation triggered by inflammatory mediators secreted from infected vaginal epithelial cells and resident immune cells (1, 45). In this study, the phosphorylation of EGFR and neutrophil recruitment appeared in vaginal mucosa in mouse VVC model and blocking EGFR or p38 signaling significantly reduced vaginal neutrophil recruitment, fungal burden, and inflammatory cytokines production induced by *C. albicans*. These results are contradictory with an observation with oral epithelial cells, in which EGFR-mediated immune responses and neutrophil recruitments protect the host against oral *C. albicans* infection (20). Nevertheless, in terms of the effects of p38 inhibition, our finding is consistent with the results from myeloid cells, in which p38γ/p38δ deletion protects against *C. albicans* infection and decreased host damage *in vivo* (46). The data again confirm that activation of the EGFR/p38 pathway is the key event to causing those deleterious inflammatory episodes and neutrophil recruitment during VVC.

Compared to drug-susceptible *C. albicans* infection, drug-resistant strain showed stronger phosphorylation of EGFR and p38 and lower ERK1/2 and c-Fos. The mechanism could be due to the differently-secreted or surface-mounted fungal ligands between two fungal strains for activating EGFR, for example hyphal-derived ligands, which is supported by better developing hyphae in drug-resistant strain in this study. The impact of p38 activation on vaginal epithelial cells is confirmed by p38 inhibitor in this study. Together with a strong positive correlation between hyphae and p38 phosphorylation in symptomatic VVC patients and lower levels of phosphorylated p38 in most yeast form of NAC infected cells (12), it is tempting to expect that EGFR- p38 is more specific for filamentous fungi. However, when we stimulated VK2/E6E7 cells with true hyphae of SC5314, we found that only phosphorylation of p38 was increased steadily, not EGFR or other MAPK protein (**Figure S2**). Obviously, other non-EGFR signaling could be also responsible for p38 activation in vaginal epithelial cells. Since phosphorylation levels of ERK1/2 and c-Fos occur at early stages of yeast form growth and subside at a later stage, ERK1/2 and c-Fos phosphorylation may be yeast-specific in VVC.

Both pathogen-mediated damage and host-mediated damage contribute to the outcome of an infectious disease (43, 47, 48). During VVC, epithelial damage is mediated by the host innate immune responses, and triggered by virulence factors of *Candida* species (47, 49). Our results have shown that EGFR, p38 and ERK1/2 inhibitor effectively suppress the inflammatory responses in vaginal epithelial cells to both clinical *C. albicans* and NAC species. A representative panel of NAC species has been shown to induce less damage and neutrophil recruitment than *C. albicans* (36). In this study, clinical NAC species including *C. tropicalis*, *C. glabrata*, *C. parapsilosis* and *C. auris* can trigger a stronger phosphorylation of ERK1/2 or c-Fos than *C. albicans* strains at 6 h post challenge. All our tested NAC strains were unable to form hyphae in vaginal tissue except *C. tropicalis*, which showed the strongest p38 and the least phosphorylation of ERK1/2 among the NAC species. This further supports that ERK1/2 may be yeast specific and p38 may be hyphae specific during VVC infection. In accord with the MAPK responses and hyphae formation, yeast-formed NAC strains induced less inflammatory cytokine production and filamentous fungal cells induced stronger cytokines. We note that *C. auris*, an emergent but rare pathogen of VVC (35) can induce higher levels of G-CSF and IL-1β than *C. glabrata* and *C. parapsilosis* although all of three species obstinately remain as yeast form. The precise identity of *C. auris* in immunophathology needs to be unraveled, but both G-CSF and IL-1β could lead to stronger inflammatory response. In addition, *C. auris* and *C. parapsilosis* triggered more damage to vaginal epithelial cells than *C. albicans* after 24 h post infection, indicating that such a strong virulence from each of these two NAC species was not contributed by hypha formation. Damage-driven responses in *Candida*-infected vaginal epithelial cells persisted at later stages of infection (50) and the survival curve lines of the infected VK2/E6E7 vaginal epithelial cells also indicated a species-specific damage-response to *Candida* pathogens.

In summary, the EGFR/MAPK pathway is differently activated in vaginal epithelial cells by clinical *C. albicans* and NAC species. Inhibition of EGFR markedly decreased vaginal inflammatory responses and epithelial damage induced by *C. albicans in vitro* and *in vivo*, while blocking p38 significantly reduced inflammatory cytokine production and cell damage induced by clinical *C. albicans* and NAC species. It is also worthy of note that ERK1/2 phosphorylation may be specific to the yeast morphology of *Candida* in VVC. These results indicate that targeting EGFR/MAPK pathway or the downstream signaling could be an effective therapeutic strategy against VVC caused by drug-susceptible *C. albicans*, NAC species and even drug-resistant *Candida* spp.

## Data Availability Statement

The datasets presented in this study can be found in online repositories. The name of the repository and accession number can be found below: NCBI Sequence Read Archive (PRJNA830650).

## Ethics Statement

The animal study was reviewed and approved by the Animal Study Committee of the Institute of Dermatology at CAMS.

## Author Contributions

JZ designed the experiments, analyzed data and wrote the manuscript. JZ, JP, HM, YY and XL undertook experiments. DL were involved in reviewing and revising the paper. WL and XS funded and supervised the project and provided scientific guidance. All authors have approved the final version.

## Funding

This study was financially supported by the National Natural Science Foundation of China (No. 81972949) to WL, the National Natural Science Foundation of Jiangsu Province, China (BK20191137) to XS.

## Conflict of Interest

The authors declare that the research was conducted in the absence of any commercial or financial relationships that could be construed as a potential conflict of interest.

## Publisher’s Note

All claims expressed in this article are solely those of the authors and do not necessarily represent those of their affiliated organizations, or those of the publisher, the editors and the reviewers. Any product that may be evaluated in this article, or claim that may be made by its manufacturer, is not guaranteed or endorsed by the publisher.
