# Supplementary material for: Divergent EGFR/MAPK-Mediated Immune Responses to Clinical Candida Pathogens in Vulvovaginal Candidiasis
Source: Front Immunol. 2022 May 26;13:894069. doi: 10.3389/fimmu.2022.894069 (PMC9204526; doi:10.3389/fimmu.2022.894069)
Supplement: Supplementary file 1 [file DataSheet_1.docx]

**Supplementary Datas**

**
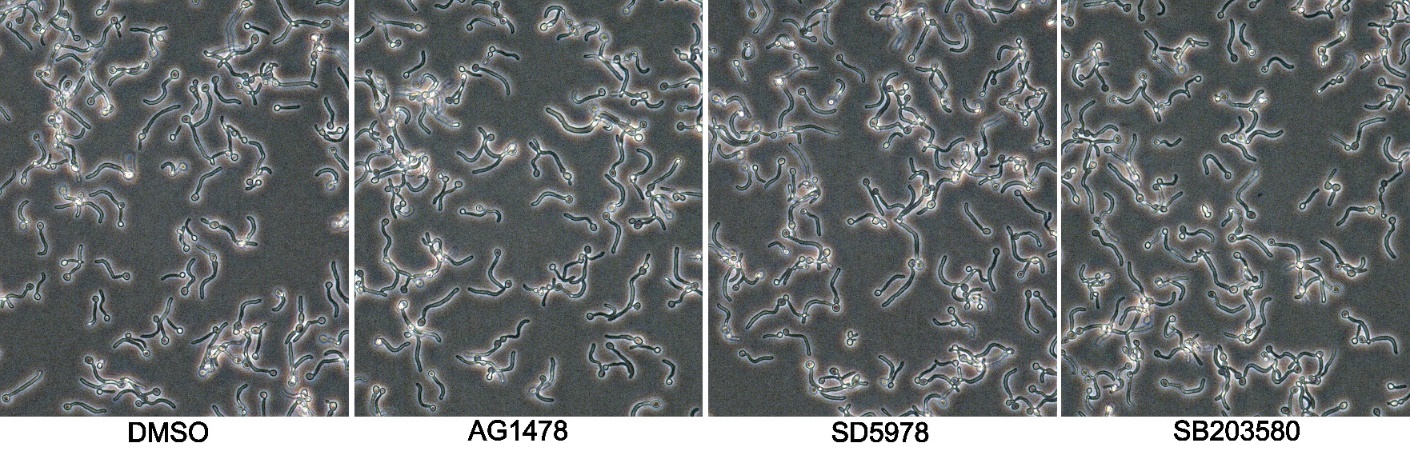
Figure S1.** No difference was observed in the formation of SC5314 hyphae at 3h post infection following pretreatment with AG1478, SD5978 or SB203580. Brightfield images taken at 400x magnification. Data are representative of data are representative of three independent experiments.

**
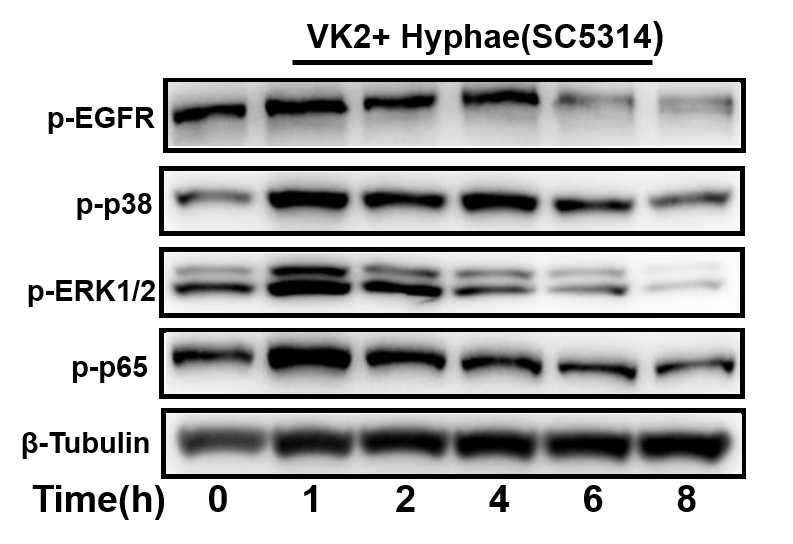
**

**Figure S2.** Induction of EGFR, p38, ERK1/2 and p65 phosphorylation in vaginal epithelial cells after infection with hyphal form of SC5314 for the indicated time. An MOI of 5 was used for all infections. Data are representative of three independent experiments.
